# Workplace-based health research training: a qualitative study of perceived needs in a rural setting

**DOI:** 10.1186/s12961-020-00580-2

**Published:** 2020-06-15

**Authors:** David Schmidt, Jill Reyment, Emma Webster, Sue Kirby, David Lyle

**Affiliations:** 1 The University of Sydney, Sydney School of Public Health, Faculty of Medicine and Health, Sydney, NSW 2006 Australia; 2 Clinical Governance, Murrumbidgee Local Health District, Wagga Wagga, NSW 2650 Australia; 3 School of Rural Health, Sydney Medical School, The University of Sydney, Dubbo, NSW 2830 Australia; 4 Centre for Primary Health Care & Equity, University of New South Wales, Sydney, Australia; 5 University Department of Rural Health (Broken Hill), Sydney Medical School, Faculty of Medicine and Health, The University of Sydney, Broken Hill, NSW 2880 Australia

**Keywords:** Rural research, research training, organisational support, research capacity

## Abstract

**Background:**

The calls for increased numbers of researchers in rural health are growing. To meet this demand, training is needed. If training is to be effective, the value placed on research, the organisational need for research training and key targets for research skill development within a rural health organisation must be understood.

**Methods:**

This qualitative study was underpinned by a critical realist perspective that allowed exploration of the organisational, cultural and structural contexts of research training and of the ability of individuals to act within these contexts. Individual interviews with purposively selected key informants from the organisation’s board, executive and facility management (*n* = 7) and two focus groups with a convenience sample of frontline health workers with interests in research (total *n* = 11) were held. Data were analysed using NVivo software and thematic analysis.

**Results:**

The themes emerging from this study were the fragmentation of research activity, a need for systems that support research and collaboration for expertise.

**Conclusions:**

This study has identified an overreliance on individual activity leading to a fragmented approach to research. There is a need for supportive structures, coordination and workplace leadership to overcome a longstanding culture that views research as out of the rural scope of practice. Identifying research training targets, partnering for educational expertise and planning for long-term sustainability are necessary steps toward increasing research activity in the longer term.

## Introduction

Rural health decision-makers and clinicians need relevant, good quality research to make improvements and enhancements to rural health services. Research capacity-building is an avenue for these organisations to have the capability to produce research that leads to better understanding of locally relevant issues and consider improvements and enhancements [1]. Research in rural areas presents its own set of challenges and the solutions proposed have included specific rural training [2] and embedded health service researchers [3]. The challenges of providing training on research skills in rural areas include a limited pool of experts in rural research, a similarly limited pool of research experts based in rural areas, structural barriers requiring thoughtful educational design [4] and maintaining support to sometimes isolated learners [5].

Several programmes have been undertaken within the rural health workspace to improve research capacity [6–13]. Some of these programmes [4, 5, 12, 13] have endeavoured to align trainee selection and research activities with the organisational priorities of the hosting organisation. However, information on the research training needs of rural health organisations on the whole is not known.

This study, conducted within a rural and outer regional portion of New South Wales, Australia, in 2016–2017, explored the perceived benefits of and perceived need for research and research training within a large rural public health organisation responsible for the management of over 40 hospitals and other health facilities over a very large geographic area. The organisation’s 3500 staff service a combination of large and small towns with significant diversity in terms of socioeconomic status and ethnicity. The region has an Aboriginal population of approximately 5%, with 9% of residents born overseas and approximately 1% non-English speaking. Health services try to work in concert with primary care providers and privately run services. Along with previous research identifying research within a rural health organisation’s strategic and operational documents [14], the results of this study will assist rural organisations in identifying the structural deficits and structural enablers that impact on the training of research skills and the conduct of research in rural areas.

This study sought to explore the perspectives of key stakeholders on the role that research plays within a rural health service, the perceived needs of the organisation in terms of research training, the perspectives on methods of training to benefit staff within the organisation as a whole and what that training should encompass, and the perspectives on which individuals within the organisation should be targeted for training in research.

For the purposes of this study, research is defined as activities that could or should be submitted to a human research ethics committee [14].

## Methods

This qualitative study was conducted using a critical realist underpinning. Realist approaches are directly applicable to organisation and management studies [15] and the use of critical realism was intended to allow an understanding of the organisation, the work culture and other ‘structures’ likely to contribute to or inhibit the ability or ‘agency’ of the individual to undertaking learning in this field [16]. These structures include not only the physical spaces of the hospitals and community-based facilities but also the cultural norms of the organisation, the policy and procedural frameworks within which research training may occur, and the financial or organisational constraints affecting research training and activity. Critical realism was the lens that was used to reveal these structures and draw inferences about the ability of these structures as inhibitors or enablers of individual agency of rural health workers wishing to undertake further training in research.

The study also explored how individuals access education and training in research or, if this is not possible, the perceived barriers to access.

Participants included the following key informants or groups: (1) members of the organisation’s board or executive with a strategic or organisational responsibility for research; (2) managers of facilities in which research is active; and (3) other staff with an interest in research.

Purposive sampling was used to identify and recruit potential board, executive and management participants. Individuals were invited by email contact from the lead researcher (DS).

Clinicians and other health staff were recruited by convenience sampling via an email flyer distributed across the organisation, inviting participation in a focus group.

Interviews were conducted either face-to-face in a location chosen by the interviewee or via telephone. One focus group was held in a meeting room at a large regional hospital whilst the other was held via teleconference. All participants provided written consent prior to interview or focus group participation.

A semi-structured approach was used for interviews and focus groups, with a pre-determined schedule of questions providing the outline and prompt questions used for further exploration of ideas and concepts. Questions were derived by the lead researcher (DS), informed by existing research capacity-building literature and refined by the other members of the research team, aimed at improving clarity and flow. No pilot testing of the questions occurred. All interviews and focus groups were digitally recorded and transcribed professionally.

Interviews lasted between 18 and 48 minutes, while focus groups lasted between 53 and 72 minutes. The lead researcher (DS) convened all focus groups and conducted interviews, at the conclusion of which field notes and reflections on the information discussed were made to inform analysis.

Data collection was concluded after all available interviews and focus groups were conducted. No attempt was made to ensure thematic saturation or redundancy as a result. Participation in the research was voluntary and individual interview participation was only known to the lead researcher and the participant. Involvement in focus groups was known to the other participants in the focus group. Participants were given the opportunity to request a copy of the final transcript; however, no participants made this request.

Thematic analysis was used with initial codes manually derived by the lead researcher by an inductive process of multiple readings of the transcripts. NVivo software (QSR International Pty Ltd. Version 10, http://www.qsrinternational.com/) was used for initial management of the data, with manual coding and theme derivation completed using the cut and paste method [17]. Memos and field notes assisted with theme development [18].

The veracity of themes was confirmed by co-researchers (JR, SK and EW) who independently read three de-identified transcripts and reviewed the derived themes. Co-researchers were able to posit new themes from the data; however, only minor discrepancies between the derived themes and the co-researchers’ interpretation of the transcripts were identified, which were resolved by negotiation and further refinement of the themes.

The lead researcher (DS) is studying research skill-building in the rural health workplace as a PhD student and works in a rural researcher training programme. The lead researcher attempted to bracket his own preconceptions [19], with memos and reflective discussion with supervisors used to ensure methodological and procedural rigour.

Ethical approval was granted by the Greater Western Human Research Ethics Committee (ref LNR/16/GWAHS/72), with governance approval from the hosting organisation.

## Results

A total of 18 individuals participated (14 female, 4 male) in the study, with seven individual interviews held with board members (*n* = 2), members of the organisation’s executive (*n* = 4) and operational management (*n* = 1). Eleven health staff, comprising a mix of clinical (*n* = 8) and non-clinical staff (*n* = 3), participated through focus groups (*n* = 8 face-to-face and *n* = 3 in the virtual focus group). Interviewing organisational leaders rather than including staff of various levels in focus groups was intended to allow free and open discussion of issues without perceived power imbalances. To preserve anonymity, the responses have been simply assigned an interview or focus group number.

From the analysis of the interviews and focus groups, three themes emerged, namely fragmentation of research activity, a need for systems which support research, and collaboration for expertise. These themes are explored in greater depth below.

1. Fragmentation of research activity

Currently, research is viewed as an individual activity rather than as part of a staff member’s work role or contribution to the organisation overall. This reliance on individual agency to drive research leads to research activity that is fragmented and has little connection to the organisation’s strategic direction.

“*Because people were coming and saying I want to do this and I want to do that and it's like that's got no relevance to where our strategic build is going and our workforce build and whatever.*” Focus Group 1

There is a need for supportive structures that would allow staff to align their preferred research activities to the organisation’s greater goals; this could include clear strategic direction for research and leadership for research within the organisation’s priorities.

The current perception within the organisation is that there appears to be little research activity, with low visibility of existing research.

“*It would be really good to know in the last five years or even more who has actually conducted research within* [the organisation] *… But it's hidden. We don't see it.*” Focus Group 1

This lack of visibility acts as an inhibiting structure. As a result, local research is not seen as leading clinical care or service priorities.

“*It should play a large role and it should drive a lot of clinical practice. However, I don’t believe that that's the case. I don't believe we have much capacity for research … it just is one of those things that are good in theory and we like to talk about it. But we don't actually put the infrastructure or the resources into doing it.*” Focus Group 1

One of the perceptions about the organisation is that, as a rural health service, they are poorly placed to undertake research and poorly resourced for research activity.

“[Our organisation] *has always thought that we are too rural and too poor to do research. And I think this is the thinking in most rural organisations or rural and regional organisations. But recently, say in the last 1 or 2 years, the thinking has been gradually shifting and changing.*” Interview 7

This perception has a real effect in that staff within the organisation have traditionally not pursued research activities, instead seeing research as something outside their capabilities as a rural organisation.

There is a tension within the organisation when considering appropriate targets for research training. One perception is that the way to build research activity and capacity is to start with those with an interest and desire for research, regardless of their position or status within the organisation. This egalitarian approach recognises that any individual or group within the organisation may have a viable and important research idea.

“*I think it needs to be available to any employee that … have a – a level of interest in it … it could be someone in administration, it could be clinicians, research skills can be applied in lots of different settings.*” Focus Group 2

Balancing this egalitarian view is a more pragmatic approach – that the organisation has limited resources and these should be targeted to those best placed to maximise the organisational benefits of this training. These individual targets include those with an existing teaching and training role or those with existing responsibility for research.

“*I guess the Utopia would tell you as many as possible* [receive training]*. In reality is that if that costs money you have to be selective about who does that, then I believe that the process of being selected can actually be quite discriminatory depending on where you sit in the organisation.*” Interview 4

The limited amount of funding acts as an inhibiting structure, leading to potential indecision for deciding who, if anybody, should receive opportunities in research and research education. This indecision, in conjunction with an overall fragmentation of activity and a lack of alignment with organisational strategy, makes developing a comprehensive and coordinated research training strategy more difficult.

2. A need for systems which support research
  “*If we start encouraging research then from our perspective we have to put systems in place so that people will know what is available, how to do it, what kind of support and what kind of funding is available.*” Interview 7

At present, there is a lack of systems and structures to support research within the organisation. Ideally, the organisation would have one or more employees with a diverse skillset who would be placed to help facilitate research and develop researchers.

“*I think somewhere in the district you have to identify one or two people … who has got good communication skills, good liaison skills, good problem solving skills, to work with these people that are actually doing that, to enable them to concentrate on their research.*” Interview 4

The level of knowledge about research is low within the organisation and people have been reluctant to engage with research due to a perception that research is complex, secretive and difficult, with systems that are obstructive and unhelpful.

“*… it’s a little bit too hard, it’s almost a bit like secret research business … I think there’s a little bit of a perception it’s secretive, it’s complicated and it can be, depending on what you’re doing, but the main practice doesn’t have to be.*” Interview 1

One structural support system that is required is the adequate resourcing of research activities. At present, there is little resourcing for time, funding or research equipment, which leads staff to pursue options outside of the organisation or to view research as unsupported.

“*I think the challenges for us are get the institution right, the culture right for individuals, to get the training right. Both in terms of people who do research and people who facilitate research and then we've got to get the last part which is the money.*” Interview 5

Institutional or structural barriers to research and research training create a perception that the organisation is actively discouraging budding researchers from learning about or undertaking research.

“*I think there are a number of barriers which probably I think characterise as institutional. So … how does an institution encourage people to think innovatively, to be looking for questions and to encourage it without becoming weary of change or resistant to change or uncaring about change? Sometimes I think good ideas get through the health institution by good luck rather than good management.*” Interview 5

One structural barrier is the geographical spread of the organisation. This may inhibit activity at some of the outlying sites and create a tendency for all activity to be centred on the organisation’s largest facility.

“*The other thing that is probably a little bit of a barrier for those people is because some of those people are in isolated areas. And we can talk about technology and we can talk about networking and all that but sometimes that isolation inhibits them from moving forward.*” Interview 6

“*Why are we only focusing on* [the major centre]*, that's my thing? There is so much research opportunity out there. We’ve got* [over 30] *hospitals. And in typical* [organisational] *form we are very* [major hospital] *centric.”* Focus Group 1

It is essential that supportive structures, including formal governance structures and informal supports such as mentoring, acknowledge and attempt to address these known barriers.

3. Collaboration for expertise

There is a recognition that research is a specialist skillset that requires expert knowledge and expert input that does not currently exist within the organisation. In the absence of this expertise within the organisation, there is a need to build partnerships with universities and other health organisations to access this level of skill. Thus, we can see a structural solution in the form of a partnership used to address a structural deficit in terms of research skill.

*“That’s where a partnership with a university would be beneficial, if you have got someone that has a high level of training in the best way to conduct research... because it’s not a focus* [of a rural health organisation] *…* ” Focus Group 2

This collaboration with university partners was seen as a means of securing such expertise, provided that the organisation retains ownership of the strategic direction for research.

“*That’s about setting the agenda, and if you’re not necessarily owning the research, being a part of the lead in the process of that research. Again, not being a passenger but actually being involved in driving where we are going.*” Interview 2

Any research training strategies implemented as part of a partnership with a university should acknowledge existing workplace cultures and the influence of competing priorities for the education and healthcare sectors.

The need for access to education and training saw participants recognise a place for online training but felt that face-to-face training may be more effective in terms of acceptance.

“*I think face-to-face, project-based and over a period of time rather than a one-off. Project-based is … a lot more engaging and they can actually practice the skills as they go, plus they get encouraged to identify a project and then the training is about supporting them to work through that and they get the practical skills rather than just - when they learn about it in a classroom environment*.” Interview 3

This approach can build on existing quality improvement and project skills already in the workforce.

*“We’re very good at project work. … But I think research is just that next stage on … It's just taking it that step – the project one step forward isn't it?*” Focus Group 1

## Discussion

This study has examined the viewpoints of rural health decision-makers and clinicians within the organisation regarding research and research training. The use of a critical realist framework allows particular examination of supportive structures and the agency of individuals to work within these structures by specifically identifying these formal and informal structures. Using this lens, it can be deduced that the organisation’s current position on research is fragmented due to a combination of limited resourcing, a lack of supportive structures and limited expertise.

At present, the organisation relies heavily on the motivation and agency of individuals with an interest in research for research activity. However, individual agency by itself cannot overcome all the structural limitations identified, including a lack of visibility, a lack of strategic leadership, cumbersome systems and poor coordination. Geographic distances between sites and an emphasis on research centred on the organisation’s largest hospital was also an issue. This centralised approach appears to inhibit research development in outlying sites. Previous research has identified that geographic barriers may be addressed instead by decentralising training [4]. It is important to recognise these barriers can exist at the individual, team and supra-organisational levels [20] as well as the organisational levels identified in this study.

Existing research training options available to staff are not well advertised and there was limited awareness of some public sector training options [5, 12, 13]. Those with an interest in learning about research are doing so via external institutions as research higher degrees, which may increase individual skill but limits the organisational benefit of this activity as there is a limited opportunity to align these projects with organisational goals; this alignment should provide a supportive structure in which individuals can receive support. While research is seen as an individual preference or activity, it remains external to the organisation’s activities and priorities. This individual motivation is seen as important for research completion [21, 22], although this is not evidenced in rural areas [5]. Individuals undertaking research education via universities are given little encouragement to subsequently use these skills within the organisation, demonstrating evidence of a cultural structure that inhibits individual agency.

The concept of a research supportive culture is difficult to define and demonstrate [14]; however, greater promotion of research endeavours [23] is one measure. Addressing fragmentation of research efforts and publicising research education opportunities in conjunction with external partners would be meaningful steps towards a research-supportive culture in this organisation.

There was a recognised need for an introductory level of research education. Some participants were keen to build on existing skills in project work or quality improvement, while others discussed engaging in cross-institutional research partnerships. This diversity of perceptions and ambitions aligns with what is known about the indicators of successful research capacity-building, with training at a level to meet individual needs, which may mean multiple levels of training are required within the one organisation [23].

Partnership between health services and universities is seen as a critical way for the organisation to engage research expertise [14] and this is known to be a way to improve research capacity [4, 20, 23, 24]. The vision of this partnership was multifaceted; a collaboration centred on a notion of reciprocity, a need for the organisation to retain control of the direction of research activity and ownership of the resultant research all featured. While expertise may need to be sourced from outside the organisation, the training must be visible within the workplace if it is to engage the workforce in a meaningful way. While there is a known link between ‘close to practice’ research as an enabler of research capacity-building [12, 20, 23], the concept of keeping research training support close to the workplace is one that is challenging for rural organisations and requires careful structural design for training programmes [4]. This close-to-practice approach also creates a tension for decision-makers at the organisational level who are responsible for determining the organisation’s research priorities, which may potentially exclude relevant ideas from smaller communities or useful ideas from individual clinicians if those fall outside the big-picture priorities.

While staff at several levels of the organisation’s leadership were approached, only a single operational manager agreed to participate. This limited engagement at the operational management level aligns with emerging research on the research capacity and culture of NSW rural health organisations [25] and the known operational responsibility for research within the organisation [14]. The use of purposive sampling at the leadership level targeted those with existing organisational responsibilities for research, which may have led to responses that reinforced an existing organisational position. Other leaders in the organisation may have presented an alternate vision for research.

There was limited engagement from smaller facilities within the organisation which may reflect issues with study design, an underlying belief that research is only for larger centres, a lack of operational responsibility for research in smaller centres [14] or the busyness of rural practice.

The lead researcher’s knowledge of research training in a rural area informed the design of the study and allowed for nuanced exploration of issues in the interview and focus group process. This knowledge lent credibility to the retroductive process of exploring ‘why things are as they are’ using critical realism [15].

While this study included only a single rural organisation, this organisation is typical of many large rural health services and, as such, the findings of this study may have a wider applicability.

## Conclusion

This study has shown that research plays a limited role within the organisation and there is a need for research training that is both introductory and supported by external expertise. While there are multiple targets for training in research, this training needs to be supplemented with a range of supportive structures to ensure improved access to information, coordination of research activity, alignment with strategic goals and sustainability.

The organisation aspires increased research capability and activity. Changing perceptions toward research, leadership, ownership and valuing of research endeavours will be key components in the longer term to reach this aspiration.

## Data Availability

The datasets generated and/or analysed during the current study are not publicly available due to privacy provisions but are available from the corresponding author on approval from the authorising ethics committee.
